# Disturbance–diversity models: what do they really predict and how are they tested?

**DOI:** 10.1098/rspb.2011.2620

**Published:** 2012-02-01

**Authors:** J. Robin Svensson, Mats Lindegarth, Per R. Jonsson, Henrik Pavia

**Affiliations:** Department of Marine Ecology, University of Gothenburg, Tjärnö, Strömstad 452 96, Sweden

**Keywords:** disturbance, richness, evenness, diversity indices

## Abstract

The intermediate disturbance hypothesis (IDH) and the dynamic equilibrium model (DEM) are influential theories in ecology. The IDH predicts large species numbers at intermediate levels of disturbance and the DEM predicts that the effect of disturbance depends on the level of productivity. However, various indices of diversity are considered more commonly than the predicted number of species in tests of the hypotheses. This issue reaches beyond the scientific community as the predictions of the IDH and the DEM are used in the management of national parks and reserves. In order to compare responses with disturbance among measures of biodiversity, we used two different approaches of mathematical modelling and conducted an extensive meta-analysis. Two-thirds of the surveyed studies present different results for different diversity measures. Accordingly, the meta-analysis showed a narrow range of negative quadratic regression components for richness, but not evenness. Also, the two models support the IDH and the DEM, respectively, when biodiversity is measured as species richness, but predict evenness to increase with increasing disturbance, for all levels of productivity. Consequently, studies that use compound indices of diversity should present logical arguments, *a priori*, to why a specific index of diversity should peak in response to disturbance.

## 1. Introduction

The well-known intermediate disturbance hypothesis (IDH) [1] and the dynamic equilibrium model (DEM) [2–4] together constitute an influential framework in ecological theory as well as in conservation and management [5,6]. The works by Connell [1] and Huston [2] have received more than 3300 and 1500 citations, respectively, and still generate important scientific papers at an increasing rate (Thomson Reuters Web of knowledge). The origin of the IDH is, however, debated [7] and can be traced back to earlier work by Eggeling [8], Odum [9] and Horn [10]. More specifically, the characteristic underlying mechanisms of the IDH was described already by Grime [11] and Osman [12], who have also received 655 and 396 citations, respectively (Web of knowledge). The IDH has been evaluated by mathematical modelling [13], and supported in laboratory studies [14] as well as in field studies in terrestrial [15], freshwater [16] and marine communities [17]. Similarly, the DEM has been supported in experimental studies in both aquatic and terrestrial systems [18–21]. The IDH and the DEM have, however, also received criticism, e.g. for being too simplistic, in both empirical and theoretical studies [22–24]. Furthermore, Violle *et al.* [25] showed that high levels of disturbance do not negate the importance of competition and Miller *et al.* [26] have identified coexistence regions for not only peaked, but also increasing and U-shaped relationships between diversity and increasing frequency and intensity of disturbance. Nonetheless, the IDH and the DEM are still used as important tools in ecological science and management, and the models generate scientific papers at an increasing rate, i.e. over one-third of the citations come from articles that were published during the last 5 years (e.g. 2006–2010; Web of knowledge).

The original formulation of the IDH predicts diversity to peak at an intermediate level of disturbance owing to coexistence of competitive dominants and rapid colonizers, while diversity will be low at both extremes owing to competitive exclusion and local extinction. Although the original, most cited, paper [1] is not completely explicit on the issue, it appears clear that the IDH is primarily concerned with richness, i.e. the number of species. There is however another aspect of diversity: the relative abundance of species, evenness, which is also of great interest for the structure and function of biological assemblages. Despite the fact that richness and evenness are two important aspects of diversity, it is not obvious that both respond in a similar way to varying intensities of disturbance. Nevertheless, predictions of the IDH are frequently and seemingly arbitrarily tested with a range of measures of richness, evenness (i.e. Pielou's evenness, equation (2.5); [27]) and combinations thereof (e.g. Margalef's richness, Simpson's *D*, 1-lambda and the more well-known Shannon index *H*′, a.k.a. the Shannon–Wiener or Shannon–Weaver index, equation (2.6); [28,29]). This use of various indices of diversity in connection with the IDH, means that the scope of the IDH has been implicitly extended to aspects of diversity for which it has no clear logical or mathematical basis. This has happened without any published mechanistic analysis or empirical justification for the use of different indices in tests of the IDH.

One possible explanation for the rich diversity of indices that are used in tests of the IDH is that related disturbance–diversity models are less clear about what aspects of diversity are predicted to change in response to disturbance. The dynamic equilibrium hypothesis (DEM) [2,3] predicts that the level of disturbance where maximum diversity is observed will depend on the level of productivity. This is because a strong disturbance is required to counteract competitive exclusions at high rates of growth, i.e. high level of productivity, whereas at lower growth rates a relatively weak disturbance is sufficient to prevent competitive exclusion. Hence, at intermediate levels of productivity, the predictions of the IDH and DEM overlap as maximum diversity is predicted at intermediate levels of disturbance. In the article where the DEM is proposed, Huston [2] defines diversity as only richness and evenness, rejecting various diversity indices, but makes no distinction in predictions between effects of disturbance on richness and evenness. Kondoh [3] discusses only species richness and does not consider specific effects of productivity and disturbance on evenness in his elaboration of the DEM. In an extension of the IDH, on differences in effects depending on the distribution of disturbance, Miller [30] stated that the highest diversity will occur at an intermediate rate of disturbance ‘…if diversity is a measure of both species abundance and number’. The addition of species abundance to the hypothesis is, however, not explained or motivated in the article. The only articles to our knowledge that discuss the relevance of different diversity measures in tests of the IDH are those by Sommer [31] and Weithoff *et al.* [32]. Both articles mainly concern phytoplankton communities and Weithoff *et al.* [32] finds functional diversity, rather than species diversity, to be the most suitable response variable for the system under study. Sommer [31] points out that theories about coexistence principally predict changes in the number of species, and not changes in relative abundances or compound indices of diversity.

Considering the large body of literature on the IDH, DEM and related models on disturbance, it is surprising that there is almost no discussion on what aspects of species diversity should be addressed (but see [31,32]). This is even more remarkable given that many other aspects of the IDH have received ample attention, such as alternative mechanisms underlying coexistence [23], influence of characteristics of communities [33], interactive effects of disturbances [34], specific traits of individual species [35], temporal variation of disturbance [36], how disturbance is applied [37] and measured [26], as well as the important discussion on definitions of ecological disturbance [38]. In contrast, explicit discussions of how to measure diversity for appropriate tests of the IDH, as well as the DEM, are lacking in even the most extensive and influential reviews on disturbance [22,39–41]. Even though the original formulation of the IDH may be straightforward, subsequent tests of the models are not and the use of various indices of diversity can be a large source of variation in outcomes among studies. Furthermore, as predictions of the DEM and the IDH overlap at intermediate levels of productivity [3], possible biases owing to the choice of response variables inevitably also concern the DEM as well as the extensions of the IDH [30]. Hence, there is much to be gained from elucidating possible differences in outcomes of tests among measures of diversity.

In this study, we contrast the response of different measures of diversity to disturbance in order to show that the measures of diversity used in tests of the IDH and the DEM are not interchangeable. We first show that models of both the IDH and the DEM generate qualitatively different predictions for different biodiversity measures. Specifically, we contrasted the two major components of diversity, which are generally combined in diversity indices, species richness and evenness. Secondly, we apply a meta-analysis complemented by a survey of the published tests of IDH to show that support of IDH indeed depends on how diversity is measured. Finally, we discuss the need for hypotheses about mechanisms explaining the relationship between magnitude of disturbance and specific measures of biodiversity.

## 2. Material and methods

### (a) Model predictions of how disturbance affects species richness and evenness

Two different approaches of mathematical modelling were used in this study. One spatially explicit model (A) on differences among measures of diversity in response to disturbance (i.e. the IDH) and one spatially implicit model (B) on the responses of diversity to interactive effects of disturbance and productivity (i.e. the DEM). Both models involve one-sided competition (i.e. species *i* competitively excludes species *j* if *i* < *j*; [3]), occupancy as a function of colonization ability, competitive strength and local extinction, which increases with disturbance. A pool of 20 species was used in all modelling runs and colonization rates of the *i*th species, *c_i_*, were modelled as *c_i_* = 0.1/0.9*^i^*: [3]. As spatial relationships are well-known to affect population and community dynamics [42,43], the first modelling approach (A) was spatially explicit using a cellular automaton model [44,45]. The model was set up as a one-dimensional universe with 100 cells. At each time step, a proportion of the cells were subjected to a random, local extinction. More specifically, at disturbance level 0.5 each cell had a 50 per cent chance of being cleared and at disturbance level 1, all cells (100%) were cleared before the colonization event. Thereafter, transition of each cell was achieved either by competition or by recruitment. In the event of competition, the state (i.e. the occupying species), *s*, of the *j*th cell at time *t* + 1, was determined by the state of neighbouring cells (i.e. competitive ability), *a*, by:2.1
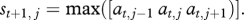
Recruitment occurred with a probability of 0.1 in unoccupied cells. The probability of recruitment of the *i*th species was modelled as:2.2
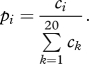
The second model (B) is a spatially implicit patch-occupancy model proposed by Kondoh [3] and later used by Worm *et al.* [18]. The model was solved using an ordinary differential equation solver in Matlab v. 7.6 (MathWorks Inc). Similar to model A, colonization rates of the *i*th species, *c_i_*, was modelled as *c_i_* = 0.1/0.9*^i^*, the extinction rate, *m*, in the model was set to 0.05 and the threshold for local extinction was 0.01 [3]. In the graphical presentations, disturbance level 1 refers to an extinction risk of 65 per cent for each species at each time step. Interspecific competition was modelled as species *i* always excludes species *j* if *i* < *j*. We modelled six levels of productivity—0.6, 2, 4, 6, 8 and 10—which covered the entire range of disturbance responses from monotonic negative to monotonic positive. Productivity *R*, and disturbance *D*, increase the rates of colonization and extinction, respectively, and the proportion *p_i_* of patches occupied by species *i* is modelled as [3]:2.3
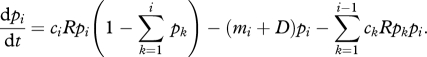


### (b) Meta-analysis of diversity measures and support for intermediate disturbance hypothesis

The meta-analysis consists of two parts. First, a survey of outcomes for different measures of diversity from studies reporting support for the IDH. Second, a formal meta-analysis that specifically contrasts quadratic regression components between richness and evenness from studies that use both measures. We specifically chose to focus on the IDH because it is the most well-cited and empirically tested among disturbance–diversity hypotheses and it therefore has the required amount of data from previously published tests to allow for meta-analyses. Furthermore, because tests of the DEM with more than two levels of disturbance that use multiple measures of diversity are not common and information on the level of productivity in studies on the IDH are rarely given, the importance of the response variable for interactive effects of disturbance and productivity could only be evaluated by the mathematical modelling. In the survey of previous tests of IDH and choice of diversity measure, we followed the methods by Shea *et al.* [22]. More specifically, we only included studies that report support for the IDH, excluded studies on abundance of single species and studies that only use two levels of disturbance. The reason for only including studies that report support for the IDH was to be able to evaluate how differences among diversity measures affect not all possible patterns between disturbance and diversity, but specifically those that are vital for the outcome of tests of well-known hypotheses (i.e. the IDH, certain level predictions of the DEM and their related models). We proceeded from the list of papers provided in Shea *et al.* [22] and complemented it by searching in Web of Science for recent articles (2003–2010) citing Connell's original paper [1]. Of the over 1000 articles initially reviewed, 160 studies in 132 publications were found which reported support for the IDH (electronic supplementary material, appendix A). Among these, 60 studies included more than one measure of diversity, mainly species richness (the number of species in a community; *S*), Shannon's index *H*′ (equations (2.4) and (2.5); [28,29]) and evenness (equation (2.6); [27])2.4
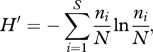
2.5
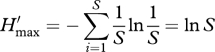
2.6
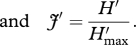
In the meta-analysis, we specifically contrasted the number of species to the evenness of species distributions in outcomes of studies that report support for the IDH using these two measures of diversity. This was done because (i) these two measures are the key components in all indices of diversity and (ii) they represent two very different components of the concept of diversity. In order to compare differences in outcomes between species richness and evenness, we calculated the quadratic coefficient in regression models describing the relationship between disturbance and richness. The quadratic components were calculated through regression analyses after *z*-transformation of data extracted from publications using the graph digitizer GrabIt (Datatrend Software, Raleigh, NC, USA). The *z*-transformations were done in order to allow comparisons between component values for richness and evenness. Disturbance levels were normalized between 0 and 1. Data extraction was possible in 28 studies from the articles reviewed (electronic supplementary material, appendix A). The strength and sign of the quadratic coefficient were then plotted with species richness on the *x*-axis and evenness on the *y*-axis. A high negative quadratic coefficient indicates a strong hump-shaped relationship between disturbance and diversity, thus supporting the IDH.

## 3. Results

### (a) Model predictions of how disturbance affects species richness and evenness

**Figure 1.**
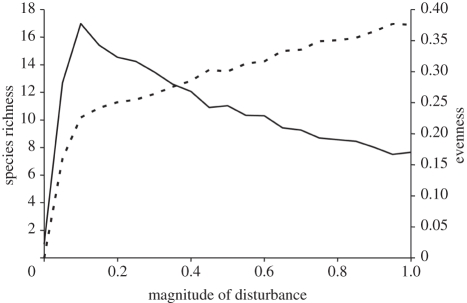
Species richness (solid line) and evenness *J*′ (dashed line) as functions of magnitude of disturbance predicted by the spatially explicit model A. Disturbance level 1 refers to all cells (100%) being cleared prior to colonization at each time step.

**Figure 2.**
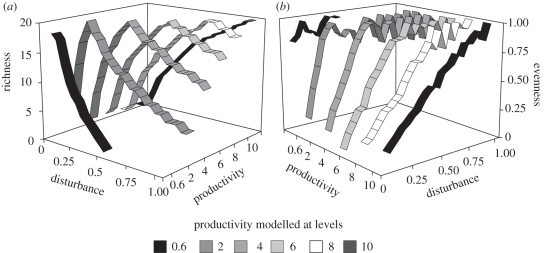
(*a*) Species richness and (*b*) evenness *J*′ as functions of levels of disturbance and productivity predicted by the spatially implicit model B. Parameters in B are: productivity levels = 0.6, 2, 4, 6, 8 and 10, extinction rate = 0.05, threshold for local extinction = 0.01, time steps = 500. Disturbance level 1 refers to an extinction risk of 65% for each species at each time step.

We applied two different approaches to mathematical modelling to explore how disturbance affects different measures of biodiversity. One spatially explicit model (A) on the effects of disturbance on diversity (i.e. the IDH) and one well-established [3,18] spatially implicit model (B) on effects of disturbance on diversity at different levels of productivity (i.e. the DEM). Here, we report effects on species richness and Pielou's evenness, *J*′ (equation (2.6); [27]), as these measures extract the two main components of species-abundance distributions. Other compound indices (e.g. Shannon's *H*′) yielded intermediate results. Both models involve one-sided competition, and occupancy of a particular species is a function of colonization ability, competitive strength and local extinction, which increases with disturbance (see §2). In model A, richness shows a unimodal hump-shaped pattern, whereas evenness is asymptotically increasing with increasing disturbance levels (figure 1). In model B, the full range of responses of richness to disturbance is shown at different levels of productivity (figure 2*a*). At low levels of productivity, richness declines monotonically with increasing disturbance; at high levels of productivity, richness increases monotonically with increasing disturbance and maximum diversity is observed at intermediate levels of disturbance and productivity, as predicted by the DEM. Surprisingly, evenness is increasing with increasing disturbance for all levels of productivity (figure 2*b*). At high levels of productivity, evenness shows a monotonic increase with increasing disturbance, whereas the increase in evenness is asymptotic at lower levels of productivity. Thus, both mathematical models predict qualitatively different effects on species richness and evenness.

### (b) Meta-analysis and survey of diversity measures and support for intermediate disturbance hypothesis

**Figure 3.**
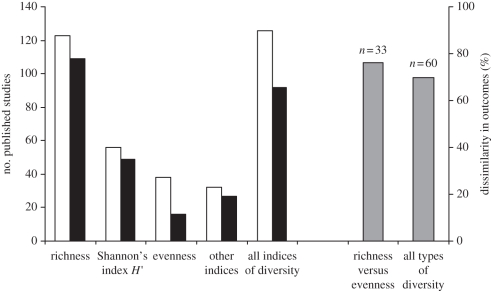
Number of studies that support the IDH for the different measures of diversity that are used in tests (primary *y*-axis), as well as dissimilarity in outcomes among measures within studies that have used two or more measures of diversity in tests of the IDH (secondary *y*-axis). White bars, tested; black bars, supported; grey bars, dissimilarity.

**Figure 4.**
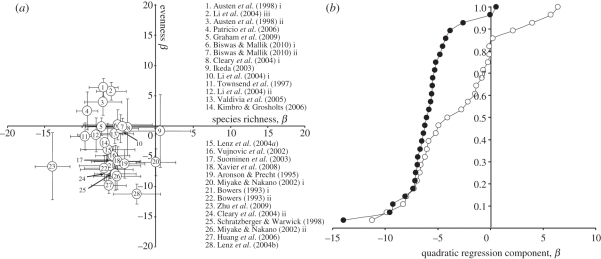
Quadratic components for species richness and evenness, calculated through regression analyses after *z*-transformation of data extracted from studies in the meta-analysis that used both measures (see §2). (*a*) Quadratic components for richness and evenness are plotted together for each study for comparisons within studies, and (*b*) the cumulative distributions of the quadratic components are plotted separately for general comparisons among measures. Support for the IDH, i.e. a hump-shaped relationship, is indicated by high negative values of quadratic components for each measure of diversity. Black circles, species richness; white circles, evenness.

Of the over 1000 articles initially reviewed, 160 studies in 132 publications reported support for the IDH and 60 of these studies included more than one measure of diversity (figure 3). In the literature survey, there were more studies, in total, that tested the IDH using various indices of diversity as the response variable, than there were studies that used species richness. When comparing single measures of diversity, species richness was still the most common measure, followed by Shannon's *H*′ and evenness. In studies that included more than one measure of diversity, the support for the IDH was often inconsistent between different diversity measures. When outcomes among all measures are compared, they show dissimilar support in 70 per cent of the cases (figure 3). In comparisons specifically contrasting outcomes among tests using both richness and evenness, these two measures differed in their support in over 75 per cent of the cases (figure 3). The outcome of the meta-analysis on quadratic regression components from the 28 previous studies that support the IDH and use both species richness and evenness as biodiversity measures is shown in figure 4. Negative values of the quadratic component in the statistical model of the effect of disturbance on diversity indicate a hump-shaped (unimodal peak) relationship and thus support for the IDH. Only when diversity is measured as species richness is there a consistent hump-shaped relation supporting IDH (figure 4*a*), and the cumulative distributions in figure 4*b* show that the range of the quadratic coefficients is narrower for the tests using species richness compared with when evenness is used.

## 4. Discussion

We here show that an established model on the DEM and a new, spatially explicit model on the IDH only show the predicted patterns when biodiversity is measured as species richness. Both models predict that evenness instead increases, monotonically or asymptotically, with increasing levels of disturbance, regardless of the level of productivity. Our extensive meta-analysis of published empirical tests of the IDH is also consistent with the model predictions as species richness yielded stronger hump-shaped relationships between disturbance and diversity than did evenness. This also corresponds with the outcome of the literature survey, revealing that two-thirds of the published studies supporting the IDH present different results for different diversity measures. Specifically, when both species richness and evenness were used, the relationship between disturbance and diversity showed an even higher degree of dissimilarity. The literature survey also revealed that there are more studies using various measures of diversity to test the IDH than studies that use species richness, and that evenness is the third most common measure of diversity used in tests of the IDH. Hence, a discussion on appropriate response variables for experimental tests of disturbance–diversity models is clearly justified.

It is surprising that the use of different diversity measures and implications for how to interpret tests of the IDH and the DEM has not received any previous attention. Mackey & Currie [41] reviewed tests of IDH and they found a hump-shaped relationship for species richness, the Shannon index *H*′ and evenness with disturbance in 19, 10 and 3 out of 85 analysed articles, respectively. They did not, however, discuss this discrepancy among measures of diversity or the possible causes of the different outcomes based on the selected measure of diversity. This potentially confounding factor in tests of the IDH is also neglected in the otherwise excellent review by Shea *et al.* [22], where they focus on the mechanisms of coexistence underlying the hump-shaped pattern.

Why then do different measures of diversity differ in response to disturbance? According to the original formulation of the IDH by Connell [1], it is the number of species that will increase when disturbance prevents competitive exclusion to occur and allows new species to colonize, up to a certain point when disturbance becomes too severe for species to persist [1,8,12]. Similarly, the DEM [2] predicts the amount of disturbance required to prevent exclusions to depend on the growth rate of the system, which was shown specifically for species richness in the model by Kondoh [3]. Thus, the prediction that the number of species should show a hump-shaped response to disturbance, for certain rates of growth, rests on logic arguments, and the hypothesis is easily tested with species richness as the most evident response variable. It is, however, less logical that this prediction should automatically also apply to the evenness of species' distributions. Species do not need to be more evenly distributed at intermediate disturbance just because the number of species is large. If the predictions are logical for the number of species, but not for species-abundance distributions, there is no clear reason for *H*′ to be a preferable index in disturbance studies, as has previously been suggested [18]. On a more general level, Stirling & Wilsey [46] argued that *H*′ was the best measure of diversity because it considers both the separate effects of richness and evenness and also their inter-relations. Although this may be advantageous under certain circumstances, it may be less so in efforts to unravel specific changes in diversity, because the underlying ecological process or mechanism causing changes in *H*′ can be traced back to effects on either richness or evenness [47]. Thus, a more interesting and challenging question is why patterns of richness and evenness differ, and if a logical pattern between evenness and disturbance can be conceived within the framework of the IDH and the DEM.

The IDH relies on the assumption that one or a few species will dominate the community in the absence of disturbance [33,48,49] and the DEM similarly predicts this to occur at intermediate to high levels of productivity [2,3]. An uneven distribution of species is therefore to be expected at low levels of disturbance, which is also commonly observed in marine and terrestrial field experiments [8,50–52]. According to the compensatory mortality hypothesis [53], mortality from causes unrelated to the competitive interactions falls heaviest on whichever species that ranks highest in competitive ability. The reduction of a highly abundant basal species (i.e. dominant) by disturbance may lead to colonization of new species in the free space [1]. Consequently, both the number of species and the evenness of species distributions are likely to initially increase following a disturbance in an already uneven community. Similarly, although in a different context, evenness has been shown to increase with herbivory in a meta-analysis on consumer versus resource control of producer diversity by Hillebrand *et al.* [54]. Accordingly, increases in evenness with increasing disturbance is shown by both the model of the IDH and for all levels of productivity by the model of the DEM (figures 1 and 2), as well as by previous field experiments from both marine and terrestrial systems [55,56].

Following the plausible increase in evenness from low to intermediate disturbance levels, logical predictions and patterns for evenness at high levels of disturbance are less clear. Commonly, high disturbance is associated with larger areas of free substratum [30,57,58]. This hinders dominants to achieve large abundances, or even exist, and allows the few rapid colonizers able to withstand the disturbance to settle in the free space. These colonizers are all likely to initially be low in abundance, which might lead to a high level of evenness despite low total coverage in assemblages at high levels of disturbance [55], which is in accordance with our model predictions. Interestingly, evenness never decreases in either model A or in model B after reaching the asymptote, regardless of the level of productivity. The lack of any decline in evenness indicates that species richness and evenness do not respond uniformly to ecological processes, i.e. disturbance and productivity. Although not in the context of disturbance, Ma [59] showed that richness and evenness of plants in experimental meadow plots were affected by different ecological processes (e.g. levels of phosphorous and nitrogen, respectively) and should therefore be considered separately in studies on diversity. Another example comes from a study on prairie microcosm communities by Wilsey & Stirling [60], in which evenness was highly influenced by species interactions, i.e. competition, while richness was influenced by the number of emerging seedlings. Similar discrepancies were found by Symonds & Johnson [61] in a study on birds, where ‘actual evotranspiration’ was the best predictor for richness, whereas evenness was best predicted by the degree of vegetation cover. They also found that there was a negative relationship between richness and evenness [61], adding to the long and ongoing debate of the possible dependence of evenness on richness [46,47,62,63]. It has been argued that the by far most common measure of evenness, *J*′ (equation (2.6) [27]), is expected to be positively correlated to richness for purely mathematical reasons [63,64]. However, an extensive meta-analysis showed that evenness *J*′ was negatively, positively or non-significantly correlated to richness depending on what group of species is examined [46]. Interestingly, McArt *et al.* [65] showed that the relationships between richness, evenness (calculated as *E*_var_) and *H*′ for arthropods is determined by the genotype of the host plant. Hence, it is evident that the possible dependences between different measures of diversity need further attention, but this is not the aim of our study. That evenness and richness have been shown to be affected by different ecological processes [46,59,61] clearly strengthen our view that these measures of diversity are not interchangeable in tests of the IDH and the DEM.

Nonetheless, despite the lack of clear working hypotheses, maximum evenness at intermediate levels of disturbance has been found in a few manipulative experiments [50,51]. Logical arguments explaining the subsequent decrease in evenness are not given in these studies, possibly because clarification of patterns thought to conform to an existing model seemed redundant. One possible explanation for low evenness at high levels disturbance is caused by the dominance of a few disturbance specialists, where a well-known example is metal-tolerant grasses on soils contaminated with mine tailings [66]. Hence, it is possible that species specialized for extreme conditions are not specifically incorporated in the framework of the IDH and the DEM, as competitive exclusion is not hypothesized to occur at high levels of disturbance. On a similar note, Violle *et al.* [25] recently showed that competition is still an important process at high levels of disturbance in protist assemblages, although the microcosms in their experiments did not allow for colonization, which is an important process in natural communities and a key component in disturbance theory [1–3,12,24]. However, it has been argued that the IDH relies on a number of assumptions [48], such as the trade-off between disturbance tolerance and competition [13]. This may open up for the possibility of incorporating a mechanism of dominance through tolerance by few species at high levels of disturbance within the framework of the IDH and the DEM, as this framework has been continuously developed and improved since the late 1970s by many authors [3,13,22,40,67,68]. However, the predicted effect on diversity, and which type of diversity, that the addition of such a mechanism has must be made very clear. Nonetheless, low evenness at high levels of disturbance has so far never been hypothesized or specifically discussed by either the original model formulators, Connell [1], Huston [2] and Kondoh [3], or by the scientists who test these models using evenness as the response variable. Consequently, evenness may not be a relevant measure of diversity for evaluations of the patterns that Connell [1] and Huston [2] predicted and the results presented in this study show that evenness is instead more likely to increase at high levels of disturbance.

In conclusion, owing to the lack of discussion of what is predicted about evenness and based on the results presented in this study, we argue that evenness is not an appropriate response variable in tests of the IDH and the DEM or their later extensions. Indices of diversity generally include both the number of species and their relative distributions, which makes assessment of their suitability in tests of the models more complex and possibly confounded. Because of this, we recommend that studies aiming to evaluate the IDH and the DEM present logical arguments, *a priori*, to why the predicted pattern should be observed for the specific index of diversity chosen as response variable for the system under study. Furthermore, as the IDH is also used in the management of marine and terrestrial national reserves and parks (e.g. Yellowstone National Park, USA), a consensus on appropriate response variables would have benefits reaching beyond the scientific community.
